# 

**Published:** 1895-01

**Authors:** 


					﻿Vol. XV.
JANUARY, 1895.
NO.1>
THE
pOW(EOPATHlC pHY^lClAfl,
A Monthly Journal of Homoeopathic Materia Medica
and Clinical Medicine.
“ He it a freeman whom truth make* free, and all are tlavet betide.”
"Seek the Truth: come whence it may, cott what it util.”
BDITXD AND PUBLISH KD BY
WALTER M. JAMES, M. D.
PHILADELPHIA : ’
JSTo. 1231 LOCUST STREET.
X/Oasr x>o it.:-
ALFRED HEATH A CO., SOLX AOBHTS YOB Obkat Bbitaim,
114 Ebury Street, Eaton Square.
4®-yearly Subeoriptien, $2.SO, invariably in advance. Single number/, US etv.
Xntarad at the Philadelphia PeauOtBea aa Saoood Claaa Mall Mather.
				

